# Social Listening for Patient Experiences With Stopping Extended-Release Buprenorphine: Content Analysis of Reddit Messages

**DOI:** 10.2196/71245

**Published:** 2025-04-25

**Authors:** Michael K Gilbert, Ashlynn R Daughton, Howard D Chilcoat, Celine M Laffont, Stephanie Strafford, Angela M DeVeaugh-Geiss

**Affiliations:** 1 Booz Allen Hamilton McLean, VA United States; 2 Indivior Inc North Chesterfield, VA United States; 3 Johns Hopkins Bloomberg School of Public Health Johns Hopkins University Baltimore, MD United States

**Keywords:** buprenorphine, extended-release buprenorphine, extended-release injectable buprenorphine, buprenorphine taper, discontinuation, social media, patient experience

## Abstract

**Background:**

Discontinuation of buprenorphine as a medication for opioid use disorder (MOUD) necessitates careful tapering to reduce opioid withdrawal and relapse. With a half-life of 43-60 days, buprenorphine extended-release formulation (BUP-XR) may provide gradual taper, facilitating successful treatment discontinuation.

**Objective:**

This study sought to understand experiences with stopping BUP-XR as described on social media.

**Methods:**

Reddit records (March 2018 to August 2022) were evaluated for the presence of referents to BUP-XR using parallel criteria based on predefined keywords and subreddit context. The keyword-based search identified records mentioning “Sublocade” (or similar search strings) regardless of subreddit. The subreddit context-based criteria identified records that were created within the r/Sublocade subreddit. Records that included reference to BUP-XR were further evaluated for mentions of treatment cessation using predefined keywords. A 50% randomized sample was then selected for qualitative analysis by a single experienced subject matter expert. Records were manually coded to validate references to BUP-XR and MOUD cessation and were evaluated for additional predefined constructs related to opioid craving and withdrawal, BUP-XR and other MOUD treatment details, and other nonmedical opioid use. Emergent constructs related to cessation-related knowledge, attitudes, behaviors, and experiences were also identified as part of the data coding and annotation process.

**Results:**

Of 6082 total, 3041 records were coded and analyzed; most (n=2692, 97.4%) referenced BUP-XR, of which, 43.8% (n=1179) referenced cessation of BUP-XR as MOUD. Many individuals shared information about prior use of MOUD, primarily transmucosal buprenorphine (185 records/63 authors), including the use of BUP-XR to taper off buprenorphine. Individuals provided details of their BUP-XR treatment experiences before and following cessation. Only 102 (8.7%) records mentioned opioid withdrawal; 1 record mentioned craving. Withdrawal experiences with BUP-XR were generally described as less intense than other drugs, although at least mild withdrawal was seen as an inevitable part of cessation. Thematic analysis revealed discussions of polypharmacy with transmucosal buprenorphine and the importance of personalized treatment and patient agency.

**Conclusions:**

There was nuanced discussion of treatment cessation using BUP-XR on Reddit, with individuals describing detailed courses of treatment and treatment experiences. Few records mentioned opioid withdrawal, and when discussed, withdrawal experiences during cessation of BUP-XR were generally described as less intense than withdrawal experiences with other drugs. These results suggest that social media, such as Reddit, can be leveraged to explore perspectives on treatment and recovery among individuals with opioid use disorder. Overall, this study provides insights into real-world patient experiences with cessation of BUP-XR and is consistent with prior case series; however, more research is needed to understand the course of opioid use disorder following cessation of BUP-XR as well as other MOUD.

## Introduction

Buprenorphine is a safe and effective treatment for opioid use disorder (OUD), first approved as transmucosal formulations in France in 1995 and the United States in 2002 [1]. More recently, extended-release injectable formulations have been approved in the United States and other countries [1]. Medications for opioid use disorder (MOUD), including buprenorphine, are associated with reduced mortality, decreased rates of opioid overdose, lower acute care use, decreased opioid use, and improved quality of life [2-5].

Currently, there is no guidance regarding the optimal duration of maintenance therapy, although longer treatment duration generally results in better outcomes [6], such as a reduced risk of posttreatment adverse outcomes [4] and increased sustained abstinence and quality of life [7]. Nonetheless, many patients discontinue buprenorphine treatment shortly after initiation, often within 90 to 180 days [8-14]. Reasons for discontinuation vary [15-19], and patients describe both medically supervised and unsupervised (self) tapers [19,20].

Despite mixed results in clinical trials assessing different taper durations [21-24], current guidance favors a slow buprenorphine taper over several months to mitigate opioid withdrawal and craving [6]. Indeed, although the euphoric effects of opioids generally motivate initial use, opioid withdrawal and craving are key drivers of continued opioid use [25]. While the somatic symptoms of withdrawal typically resolve within days to weeks after cessation of opioid use, affective symptoms and craving can persist for months or even years after cessation due to profound changes in the brain resulting from chronic opioid use [25]. Therefore, those symptoms, or their anticipation, can impede successful transmucosal buprenorphine discontinuation [19,26-28]. Patients report challenges with tapering buprenorphine, particularly with the transition from 2 mg transmucosal buprenorphine to no treatment, using strategies such as cutting strips or dissolving tablets in water to achieve smaller buprenorphine doses to facilitate tapering and minimize withdrawal symptoms [29].

Sublocade is a once-monthly extended-release buprenorphine formulation (BUP-XR) approved in the United States in 2017 for the treatment of OUD. Following subcutaneous injection, buprenorphine plasma concentration peaks at approximately 24 hours postdose and then decreases to a “plateau” characterized by the slow decline in plasma levels over time [30,31]. Pharmacokinetic modelling shows that once steady-state is achieved, buprenorphine plasma concentrations remain above 2 ng/mL for 2 to 5 months on average after the last injection, depending on the dose administered (100 or 300 mg, respectively) [30,32]. Given the unique pharmacokinetics of BUP-XR and gradual decrease in buprenorphine plasma concentrations with time (half-life of 43 to 60 days), this formulation may provide an effective taper when buprenorphine treatment is discontinued, minimizing the resurgence of withdrawal symptoms and craving. The use of BUP-XR to facilitate buprenorphine discontinuation has been the subject of several recent case series [26-28].

Our study sought to understand individual experiences with stopping BUP-XR as described on Reddit, including experiences and perceptions of opioid craving and withdrawal. Specifically, this study implemented a social listening framework to interrogate and characterize experiences as articulated in publicly accessible online forums. This approach has been applied extensively in the pharmacovigilance and postmarketing surveillance domains [33-35] as well as to explore patient experiences with transmucosal buprenorphine [29]. This study does not intend to present an exhaustive or generalizable estimate of the prevalence of the constructs under investigation, but rather, a report of the findings available using the data and methods described and guidance on the definition and operationalization of constructs for further research into knowledge, attitudes, behaviors, and experiences related to the cessation of MOUD.

## Methods

### Study Design

Reddit records (March 2018 to August 2022) were gathered in December 2022 via the publicly accessible application programming interface end points for Pushshift’s collection of historical Reddit submissions and comments, referred to hereafter as “records” [36]. Reddit is a major social networking site [37], with an estimated 70 million daily active users and 100 million active subreddits (communities) as of October 2023 [38].

In December 2022, records were evaluated for the presence of referents to BUP-XR using parallel criteria based on predefined keywords and subreddit context. The keyword-based criteria identified records, regardless of subreddit, that contain case-insensitive matches to strings within a Levenshtein distance of 1 from “Sublocade” (eg, “sublocase” or “sublocades”) within their user-populated free text fields [39] (Table 1). The subreddit context-based criteria identified records that were created within the r/Sublocade subreddit, a subsection of Reddit focused on discussions of Sublocade.

**Table 1:** Dataset schema and data annotation.

Field name	Datatype (format)	Description
ID	Text	An obfuscated base-36 unique identifier of the record
Text	Text	Open text field containing the content of the “body” field for Reddit comments or the “title” and “selftext” fields for submissions
Author ID	Text	An obfuscated unique identifier for each Reddit account
Created date	Date (epoch timestamp)	An epoch-formatted timestamp representing the number of seconds elapsed between the beginning of January 1, 1970, and the creation of the post
Notes	Text	An open text field containing any notes captured during analysis, including references to useful examples, identification of novel language, or flags for further investigation
Sublocade	Binary (1/0)	Reference to Sublocade, by brand name or clear contextual indication For Sublocade Referents, criteria include: Body OR Title OR Selftext field contains one or more case-insensitive matches to the following strings: sublocade; subloccade; sublacade; sulocade; subblocade; sublucade; sublockade; sublocate; subloocade; sublcocade; sublocode; suublocade; subloclade; sublicade; sublocades; subclocade; sublocane; sublocadr; sublocase; subocade OR subreddit field equals: sublocade
Any cessation	Binary (1/0)	Any mention of cessation of Sublocade treatment. For cessation referents, criteria include: Body OR Title OR Selftext field contains one or more case-insensitive matches to the following strings: taper; stop; final; quit; last; reduce; decrease; discontinue
Match criteria	Text	Keyword(s) or record attributes that qualify record for inclusion—see also Sublocade, any cessation
Intentional cessation	Binary (1/0)	Planned or patient-directed cessation of treatment with Sublocade
Circumstantial cessation	Binary (1/0)	Unplanned or involuntary cessation of treatment with Sublocade
Within-course withdrawal	Binary (1/0)	Reported experience of symptoms attributed to reduction or absence of buprenorphine during treatment with Sublocade
Postcourse withdrawal	Binary (1/0)	Reported experience of symptoms attributed to reduction or absence of buprenorphine after cessation of treatment with Sublocade
Final dosage	Integer	Milligram of buprenorphine in the final reported Sublocade dose
Opioid craving	Binary (1/0)	Reported experience of need or desire to redose any opioid after treatment with Sublocade
Duration of MOUD^a^ treatment	Integer (months)	Total reported duration of treatment with any MOUD (buprenorphine, methadone, or naltrexone)
Duration of buprenorphine treatment	Integer (months)	Total reported duration of treatment with buprenorphine
Duration of Sublocade treatment	Integer (months)	Total reported duration of treatment with Sublocade
Course of treatment	Array (eg, “100, 300, 300, 100”)	Reported sequence of Sublocade doses
Number of doses	Integer	Reported number of Sublocade doses
Prior MOUD	Text	Reported MOUD used before Sublocade
Subsequent MOUD	Text	Reported MOUD used after Sublocade
Concurrent MOUD	Text	Reported MOUD use concurrent to Sublocade
Subsequent NMOU^b^	Text	Reported nonmedical opioid use after Sublocade
Concurrent NMOU	Text	Reported nonmedical opioid use concurrent to Sublocade

^a^MOUD: medication for opioid use disorder.

^b^NMOU: nonmedical opioid use.

Records that contained keyword- or context-based BUP-XR referents were deduplicated using an obfuscated unique identifier derived from the “ID” value that Reddit implements internally as a unique identifier for each record. No demographic data are available for a unique record “ID.” Records that contained BUP-XR referents were further evaluated for the presence of referents to BUP-XR cessation using predefined keyword-based criteria to identify records that contain case-insensitive matches to the strings presented in Table 1. Date-based filtering was applied, and records created on or between March 1, 2018 (reflecting BUP-XR’s US commercial availability [40]), and August 31, 2022, were included in the dataset.

A random sample comprising 50% of the normalized and deduplicated dataset was subjected to manual coding to validate references to BUP-XR and MOUD cessation and to identify references to the constructs defined in Table 1. Records containing referents to cessation of treatment with BUP-XR were evaluated for additional predefined constructs related to the course of treatment, including experiences of opioid craving or withdrawal; use of other opioids before, concurrently, or after treatment with BUP-XR; and any emergent themes in narrative descriptions of patients’ treatment experiences (Table 1). Records appearing before and after the target record in the primary source material were consulted for context if clarification was required to determine appropriate coding.

In addition to the analysis of the predefined constructs outlined in Table 1, a thematic analysis was performed to characterize themes in cessation-related knowledge, attitudes, behaviors, and experiences expressed in the dataset. Thematic analysis included the development of reflective notes, the definition and maintenance of a codebook capturing emergent constructs, and a rereview of data under temporal-, authorial-, and thread-based sorting structure, all conducted without input or influence from this study’s sponsor.

**Figure 1:**
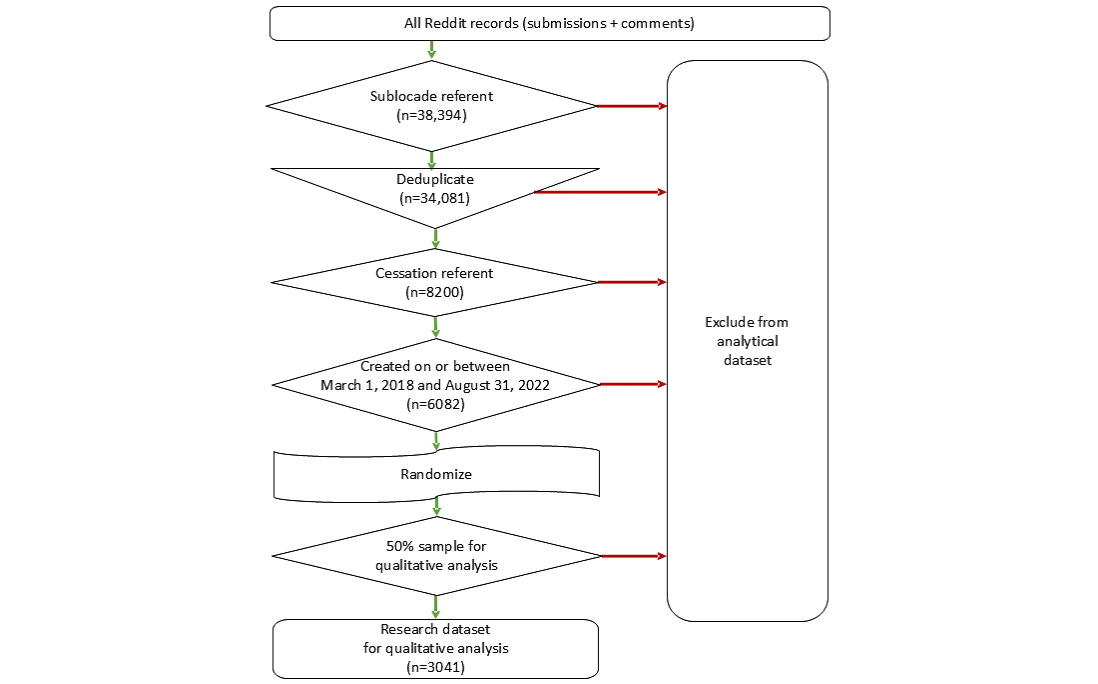
Data gathering results.

Manual coding and thematic analysis were performed by a single subject matter expert (MKG) with specialized knowledge of language and constructs prevalent in online discussions of medical and nonmedical drug use [41]. The use of a single analyst was implemented to support the efficiency of a provisional coding process as part of a broader research program with the aim of developing an initial coding scheme for use in the training of algorithmic models that could then be tuned through reinforcement learning by additional subsequent reviewers [42]. A summary of the data-gathering process described above is presented in Figure 1.

### Ethical Considerations

This study does not meet the criteria for human participant research review. All records were obtained from a publicly available website; however, because the content reviewed includes health information related to MOUD, additional steps were taken to protect privacy. Personally identifiable information was protected by identifying records only via obfuscated post and user identifiers (ie, Reddit post IDs and usernames were hashed and salted in memory as they were gathered via Pushshift). Additionally, any personally identifiable information contained within records was redacted during manual review. To further protect the contributor’s anonymity, the records described herein were paraphrased; edits did not fundamentally change the post’s content but were made in such a way as to mitigate the risk of reidentification via internet searches.

## Results

### Overview

A total of 6082 unique records satisfied this study’s inclusion criteria. A random sample of 3041 (50%) records were subjected to manual coding and thematic analysis (Figure 1). The final dataset was authored by 672 unique Reddit accounts (range: 1-275 records/account; mean 4.5 records/account, SD 13.6) and distributed across 23 subreddits, with 2968 (97.6%) occurring in the r/Sublocade subreddit.

### Manual Coding

#### Overview

Most of the records subjected to manual coding (n=2692, 97.4%) were confirmed to contain references to BUP-XR, of which 1179 (43.8%) referenced the cessation of BUP-XR as a pharmacotherapy for OUD (n=976, 82.8% described retrospective events, and n=203, 17.2% described planned events).

#### MOUD Referents and Course of Treatment

**Figure 2:**
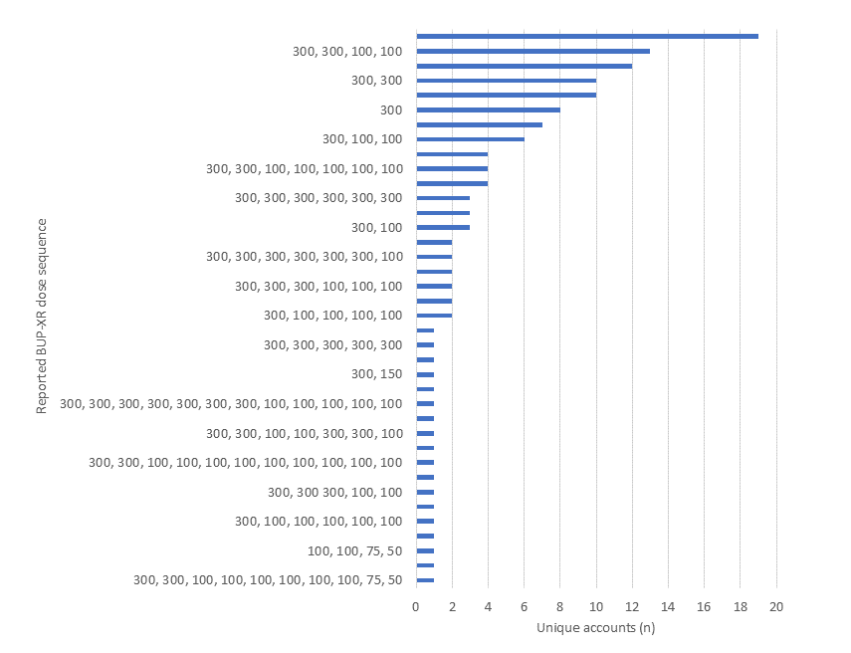
BUP-XR courses of treatment: distribution of explicitly identified BUP-XR dose sequences (N=136). BUP-XR: buprenorphine extended-release formulation.

In addition to referencing BUP-XR cessation, many described their BUP-XR course of treatment, as well as their prior use of MOUD, primarily transmucosal buprenorphine (185 records by 63 unique accounts). Specific reference was made to the number of BUP-XR doses received before cessation (mean 4, SD 2.9 doses; median or mode 3, IQR 2 doses) in 635 records by 172 unique accounts. The sequence of BUP-XR doses received before cessation was described by 136 unique accounts, including 15 individuals who reported only a single dose before cessation (8 reported a single 300 mg dose, 7 reported a single 100 mg dose; Figure 2). An additional 24 unique accounts reported only their final dose before BUP-XR cessation without detailing their full course of treatment. The most common final dose was 100 mg, reported by 127 (79.4% of 160 unique accounts reporting final dosage) unique accounts. Some (n=18 unique accounts) reported resuming MOUD after BUP-XR cessation. Among those 18 reports, 3 described the use of naltrexone, 3 described the use of transmucosal buprenorphine, 3 described the use of nonspecific “subs” or described “supplementing” with unspecified medications, and 9 described resuming BUP-XR treatment. Seven records authored by 7 unique accounts explicitly reported concurrent use of multiple MOUD, with 4 describing concurrent use of Suboxone and the remaining 3 describing the use of unspecified “supplementation” or “doses.”

#### Opioid Withdrawal and Craving

Opioid withdrawal experiences during or after cessation of BUP-XR treatment were described in 102 (8.7% of the 1179 records referencing cessation of BUP-XR) records. The articulation of opioid craving as an experience related to cessation of treatment was limited to a single record.

### Narrative Review of Opioid Withdrawal and Craving Records

Discussion of withdrawal reflected nuanced experiences and interpretations relating to symptom intensity and context of patients’ experiences, as well as interrogation related to attribution of those experiences to cessation of BUP-XR treatment. Withdrawal symptoms associated with BUP-XR cessation were most often described as less intense than those associated with cessation from other drugs or medications, with comparisons to relatively more challenging experiences after cessation of transmucosal buprenorphine or benzodiazepines.

It wasn’t nearly as bad as I had expected. Some yawning, but withdraw symptoms were minimal compared to what I had experienced before. I used to struggle to go a day without subs but Sublocade basically tapered all by itself, something I had been trying to do unsuccessfully for a year.

Some authors expressed that the symptoms associated with cessation of BUP-XR might not be appropriately characterized as “withdrawal,” based on the relatively minor impacts on affective and somatic states and the perceptions that any experiences of discomfort fell within the bounds of natural and expected variance in self-assessed wellness.

She had mild withdrawals, and not even actually withdrawals per se, she was just more tired than usual for a few months after the last shot. … That’s the reason I got on sublocade in the first place, because of her story.

Some authors described belief in the inevitability of withdrawal symptoms, including emotional symptoms or depression, asserting that such experiences were an unavoidable but manageable aspect of the buprenorphine cessation experience and were, in some cases, a return to normative emotional states or a manifestation of latent issues not experienced while on opioids. Other authors reported that they did not experience any withdrawal symptoms after cessation of treatment with BUP-XR, though these reports may reflect differences in thresholds of intensity that inform authors’ assessments and attributions of symptoms, as described above.

Only 1 record of opioid craving was identified in the 1179 records containing a reference to BUP-XR cessation.

I spend my time thinking about using or getting back on subs, I end up searching my house up and down, often looking in the same places thinking I must have missed something. For a while, I didn’t even have any cravings or urges, but lately they are intense, as intense as when I was actively using and looking for my next fix.

### Thematic Analysis

Thematic analysis identified constructs related to concomitant transmucosal buprenorphine during BUP-XR initiation and cessation, pharmacokinetics and pharmacodynamics of BUP-XR, and the influence of perceived agency and care personalization on patients’ treatment experiences.

### Concomitant Transmucosal Buprenorphine During BUP-XR Treatment

A notable theme among records referring to concomitant use of multiple MOUD formulations as part of initiation, maintenance, or cessation of pharmacotherapy related to the use of transmucosal buprenorphine products to ease the processes of initiation or cessation of treatment with BUP-XR. On this topic, many records discouraged the use of transmucosal buprenorphine as an adjunct to BUP-XR initiation, with rationales including the importance of breaking daily dosing habits and the challenges of identifying effective BUP-XR dosage with multiple products in concurrent use.

Once you have two shots there’s no physical way you could be in withdrawal and anything you take will just delay your process. That’s all a mental side effect of breaking the daily dosing habit. Especially when you’ve been tapering and cutting strips into tiny pieces, it starts to feel like active addiction and it’s something you will just have to work through.

### BUP-XR Pharmacokinetics and Pharmacodynamics

Treatment planning discussions referenced concepts of pharmacokinetics and pharmacodynamics and the Food and Drug Administration–approved label for BUP-XR as support for theories about optimal courses of treatment and expectations for the duration of effective and detectable systemic concentrations of buprenorphine. Some included explicit statements of the number of days since the last BUP-XR treatment when drug tests remained positive or when the first negative test result was obtained and often made references to aspirations for negative test results.

For the first 6 months it was strictly urine tests as I got farther from my last shot my doctor ordered blood tests because they are apparently more accurate. The levels are pretty low now, but still positive. I hope that when I go back again my results will be negative.

These conversations were marked by nuanced discussion of testing thresholds and distinctions between therapeutically effective versus assay-detectable buprenorphine concentrations, generally expressing preference and aspiration for negative drug testing results regardless of biological significance or assay sensitivity thresholds. Specific reasons were expressed by some, including a desire for negative tests in advance of planned conception, a desire for negative tests to avoid stigma or disruption in the pursuit of future employment that required drug testing, desire to simplify anesthetic and analgesic treatments for planned medical procedures, and a generalized desire to validate the absence of exogenous opioids or other drugs from their bodies.

### Patient Agency and Treatment Personalization

A prominent subtext in discussions of treatment planning was the value of personalized approaches to treatment and the importance of patients’ agency in determining their course of treatment. This was frequently invoked in discussions of partially administered doses, with authors attesting to health care providers’ willingness to collaborate in care decisions and to tailor treatment plans to better serve patients’ needs and preferences.

Experienced providers are seeing how incredible the injection is and using their own observations to figure out alternative dosing to Indivior’s ‘recommendations.’ When it comes to how to taper people off of Sublocade, we renegades are doing independent field research, just without being published or recognized.

Patients described their desire to receive partial doses upon initiation of treatment with BUP-XR to ease the transition from low-dose transmucosal medications. They also described plans and experiences of receiving partial doses or extending the interval between standard doses to ease the transition from treatment with BUP-XR to cessation of buprenorphine use.

Patient-provider relationships were also invoked in discussions of communication about cessation of treatment with BUP-XR. Some authors expressed experiences or intents of discontinuing their course of treatment without, or in contradiction of, prior communication with care providers. In many of these cases, patients described rigid clinical policies and treatment practices as incompatible with their own knowledge, needs, or intentions related to cessation. This theme of planned but uncommunicated discontinuation was particularly prominent among discussions of single dose, or “one and done,” courses of treatment. Records describing this practice often expressed this approach in contradiction of clinical guidance or care planning, with justifications rooted in patients’ desire to pursue the shortest effective course of treatment and to expedite complete cessation from MOUD.

## Discussion

### Principal Findings

This study investigated patients’ experiences with the cessation of BUP-XR as a pharmacotherapy for OUD as described on a social media platform. Data reviewed for this study reflect diverse and in-depth discussions of patient and provider knowledge, attitudes, behaviors, expectations, experiences, and outcomes associated with the discontinuation of BUP-XR. In addition to a clear articulation of case histories and other constructs that were of a priori interest to this study’s team, detailed descriptions of somatic, emotional, scientific, and political aspects of MOUD use and cessation were observed.

Less than 10% of posts mentioned experience with opioid withdrawal, and, when discussed, withdrawal symptoms were described as less intense than those associated with cessation from other drugs or medications, especially relative to previous experiences after tapering transmucosal buprenorphine. Opioid withdrawal is an important component of the cycle of addiction and includes both somatic signs (eg, aches and pains, nausea, diarrhea, or hot or cold flashes) and signs of negative affect (eg, anxiety, aversion, or anhedonia) [43,44]. While somatic signs can motivate individuals to reuse opioids to alleviate their symptoms and pain, those resolve in a few days to weeks following the discontinuation of opioid use [25]. In contrast, the affective signs of withdrawal result from profound changes in the brain’s reward circuitry and can persist for months or even years, increasing the vulnerability to craving and the probability of relapse [25,45]. A review of the neurobiological mechanisms underlying opioid withdrawal explains how neuronal changes induced by repeated cycles of opioid exposure and withdrawal intensify behaviors directed toward drugs and drug-paired cues [25]. Especially, preclinical studies have shown that the use of opioids during opioid withdrawal promotes future drug-seeking behavior and consolidates the cycle of addiction [46,47]. With their long half-life (24 to 40 hours), MOUDs such as buprenorphine and methadone provide more stable levels of mu-opioid receptor agonism, preventing the highs and lows associated with illicit opioid use; still, relapse remains a concern once MOUD treatment is discontinued [25]. By providing minimal day-to-day fluctuations in buprenorphine exposure, with plasma concentrations slowly decreasing over several months [30], BUP-XR minimizes changes in mu-opioid receptor agonism, which may explain the low incidence (and intensity) of opioid withdrawal symptoms and craving reported in this study. These results further our current understanding of patient experiences with BUP-XR discontinuation, an area of clinical importance given the limited data available to guide treatment discontinuation and reports of patient desire for, and challenges with, discontinuation of buprenorphine treatment. It should be noted, however, that the overall discussion on opioid withdrawal in our study was generally low and that the nature of these data precludes making definitive conclusions regarding BUP-XR’s role in facilitating the discontinuation of buprenorphine. Nonetheless, results align with several case series describing the successful use of BUP-XR to facilitate the discontinuation of transmucosal buprenorphine [26-28]. More research is needed to understand the success in recovery after BUP-XR discontinuation compared to standard taper approaches using transmucosal buprenorphine, as well as the ideal duration of maintenance treatment before discontinuation.

The observation of only a single reference to craving was unexpected based on its centrality in the regulatory and pharmacotherapy literature on MOUD [6,48,49]. The relative scarcity of references to craving may reflect that those contributing to the conversations gathered as part of this study may articulate their experiences using alternate language or framing. For example, authors may have framed the experience of desiring to consume additional or alternate opioids using the generalized language of “withdrawal,” or that they experience the desire to consume opioids as an obvious and unremarkable reaction to more and salient somatic experiences. These results highlight the importance of understanding and using language that is meaningful to those with lived experiences when conducting studies requiring keyword identification or when engaging with patients during treatment.

The reasons described for cessation of buprenorphine included stigma, desire for a negative buprenorphine drug test due to conception, occupational requirements, anticipated need for medical procedures, and a general desire to no longer require any exogenous opioid treatment. These reasons were consistent with prior qualitative research, in which individuals have expressed a desire to discontinue treatment due to practical or logistical issues, such as the rigidity of treatment programs and side effects, as well as concerns about being “dependent” on buprenorphine; stigma and disapproval from friends, family, or abstinence-only counseling programs; concerns about positive drug screenings at work; or a belief that they are not in recovery as long as they remain on MOUD [15,16,18,50]. Similar to prior studies [19,20], individuals described tapering off treatment both as part of active treatment planning in collaboration with their prescriber as well as without, or in contradiction of, their prior communication with their prescribers [51]. Though there are risks to discontinuing treatment, both our study and other research underscore the importance of self-empowerment and individual agency regarding treatment decisions. Understanding a patient’s motivation to discontinue treatment may provide clinicians with important insights on how to engage in shared treatment decision-making. Open conversations regarding treatment, including the risks of treatment discontinuation, may ensure that such decisions are evidence-based and approached with appropriate planning and caution.

Finally, sentiments and expectations observed in the thematic analysis suggest that research into patient experiences with BUP-XR should include engagement with patients, including online communities. Patients’ perceptions of alienation and marginalization in their relationships with clinicians, regulators, and pharmaceutical manufacturers may foster mistrust and miscommunication that could be mitigated by further studies that reflect the purposive pursuit of patient knowledge, experiences, and insights. Numerous records affirmed the value of interpersonal support received through online forums, and many cited the value of sharing their own experiences and providing support to others as valuable components of their recovery processes. Online forums were frequently described as sources of valuable information, both for peer communication and guidance, but also for postmarketing research and scientific hypothesis generation. In the context of social listening methods, this may suggest opportunities for future studies to engage people with lived experience of substance use disorder and treatment as collaborators in research activities and for research teams to proactively engage members of online communities in postresearch, knowledge-sharing sessions to facilitate integration of findings across stakeholder groups.

Altogether, our results align with prior research demonstrating that social media can be a robust source of data for potentially sensitive topics [52,53]. Studies of social media may also provide an avenue for the examination of research questions that would not be feasible using other methods. Further, studies of social media can not only shed light on patient experiences and the course of MOUD treatment, as well as other aspects of OUD, but they can also provide insights to guide the design of future quantitative as well as qualitative studies.

### Limitations

This study is subject to limitations that may limit generalizability to the broader population of patients treated with BUP-XR. It includes data from a single data source, Reddit, that may not represent the experiences of individuals who use other online platforms or who do not discuss their treatment in any online contexts. It does not include sociodemographic data on authors individually or in the aggregate, so the distribution of experiences across authors of different ages, genders, races, and socioeconomic positions is unknown. Although individuals from an estimated 212 countries visited Reddit daily in 2015 [37], these results may not be generalizable to ex-US or non–English-speaking locations. This study was designed to identify BUP-XR-specific Reddit records during the period after commercial availability in the United States. However, included records may refer to other branded long-acting injectable buprenorphine products available in Europe and Australia, although this seems unlikely given the timing, referents, and subreddit-specific search used. Further, while the content suggests that records were largely contributed by patients or individuals in treatment, it is possible that authors included health care professionals or others interested in patient care and research. Each post was attributed to a unique Reddit account; however, individuals may use multiple accounts, leading to duplication in counts aggregated by author. Manual coding and thematic analysis for this study were performed by a single reviewer with established expertise in the field of social listening for pharmacovigilance, but that reviewer’s analyses may not be exhaustive nor representative of the interpretations and syntheses that might be derived from a team of reviewers with diverse perspectives and lived experiences relevant to the data and subject matter. Nonetheless, these results provide important initial insights into patients’ experiences with discontinuing BUP-XR.

### Conclusion

A combined keyword- and context-based approach to gathering social media posts related to medications for OUD was effective in gathering posts with references to the target product. There was robust and nuanced discussion of treatment cessation among individuals treated with BUP-XR who posted on Reddit. BUP-XR withdrawal experiences were generally described as less intense than withdrawal experiences with other drugs, consistent with the long half-life of BUP-XR and the slower decrease of buprenorphine plasma levels over time after cessation. Although this study provided initial insights into patients’ experiences after stopping BUP-XR, more research is needed to understand the course of OUD following cessation of BUP-XR as well as other MOUD.

## Abbreviations

BUP-XR: buprenorphine extended-release formulation
MOUD: medication for opioid use disorder
NMOU: nonmedical opioid use
OUD: opioid use disorder

## Data Availability

The data analyzed during this study were obtained in December 2022 via the Pushshift repository [54].
